# Immune Reactions following Cord Blood Transplantations in Adults

**DOI:** 10.4061/2011/607569

**Published:** 2011-06-05

**Authors:** Hiroto Narimatsu

**Affiliations:** Advanced Molecular Epidemiology Research Institute, Faculty of Medicine, Yamagata University, 2-2-2 Iida-nishi, Yamagata 990-9585, Japan

## Abstract

Cord blood transplantation (CBT) is an attractive alternative therapy in adult patients with advanced hematological malignancies in whom matched donors are unavailable. However, the risk of complications, especially infections, post-CBT increases the mortality rates in these patients. Although the incidence of acute and chronic graft versus host disease (GVHD) post-CBT is lower than that following bone marrow transplantation and peripheral blood stem cell transplantation (SCT), the additional immunosuppressive therapy required to treat it could increase the mortality in these patients. Further, chronic GVHD following CBT is milder and responds better to treatment than that occurring after bone marrow transplants. Unlike bone marrow transplantation, the onset of GVHD is a positive prognostic indicator of overall survival in patients receiving CBT, due to the graft versus malignancy (GVM) effect. This paper focuses on the immune reactions following CBT and aims to elucidate a management strategy for acute and chronic GVHD.

## 1. Introduction

Cord blood transplantation (CBT) represents an attractive alternative for patients with advanced hematological malignancy who lack matched related or unrelated donors. Adult patients receiving myeloablative or reduced-intensity CBT display a 90% chance of engraftment, but also experience a 50% rate of transplant-related mortality, mostly attributable to infection [1–6].

Unique manifestations of immune reactions that differ from those seen in conventional allogeneic stem cell transplantation (allo-SCT) may occur after CBT. Fortunately, the incidence and severity of acute graft-versus-host disease (GVHD) after unrelated CBT are low compared with those after allo-SCT from a matched unrelated or mismatched family donor, despite infusion of human leukocyte antigen (HLA)-mismatched graft [7, 8].

The clinical characteristics of patients with chronic GVHD after CBT have not been well described. Studies dealing with CBT have mainly focused on short-term events, such as engraftment, acute GVHD, infections, and regimen-related toxicities. Several groups have reported chronic GVHD rates ranging from 17% to 89% [5, 6, 9–20]. However, studies dealing with details of the clinical characteristics of chronic GVHD, including the target organs, grading and treatment response, are limited [5, 6, 9–20].

In this paper, I focus on the characteristics of immune reactions following CBT, review the research performed to date in Japan, Europe, and the United States, and attempt to elucidate a management strategy that takes those clinical characteristics into account.

## 2. Early Immune Reactions and Acute GVHD

### 2.1. Clinical Features

Posttransplant immune disorders, including early immune reactions and acute GVHD, are potential complications following CBT in adult patients [1, 3, 21]. Such reactions and/or additional immunosuppressive therapy might increase the risk of infection and organ dysfunction, leading to high rates of transplantation-related mortality. 

Reportedly, the time course of the manifestations of early immune reactions to CBT are unique. A research group from Japan first classified early post-CBT immune reactions according to the time course following CBT as: pre-engraftment immune reactions (PIR), engraftment syndrome (ES), and acute GVHD (postengraftment immune reaction) [1, 21, 22]. ES and acute GVHD has been reported in SCT using bone marrow and peripheral blood stem cells, whereas PIR has not been well described. PIR is characterized by high-grade fever and weight gain and develops at a median of day 9 post-CBT in approximately 80% patients [21]. The immune reactions after CBT that were defined in the study by Kishi et al. [21] were used by another research group [23]. According to them, when febrile patients (body temperature ≥38°C) with no evidence of infection or adverse effects of medication exhibited skin eruptions, diarrhea, jaundice (serum total bilirubin >2.0 mg/dL), or weight gain >10% of baseline, these changes were defined as immune reactions. Reactions were classified into subtypes of pre-, peri-, and postengraftment reactions according to their timing of occurrence. Immune reactions developing ≥6 days before engraftment were defined as pre-engraftment immune reactions (PIRs), while reactions within 5 days of engraftment were defined as engraftment syndrome (ES). Other reactions were defined as postengraftment syndrome, generally corresponding to acute GVHD.

The clinical features of PIR have been characterized in both reduced-intensity conditioning CBT and myeloablative CBT. [1, 21–25]. The incidence of PIR was relatively high in studies from Japan where preparative regimen without antithymocyte globulin (ATG) coupled with cyclosporine alone as GVHD prophylaxis was mainly employed [22, 23, 26]. Thus, there is a possibility that insufficient immune suppression in CBT may play a role in the development of PIR. 

Some researchers have reported that the occurrence of PIR depends on HLA disparities between recipient and cord blood units [1, 21]. PIR supposedly develops in conjunction with a cytokine storm or homeostasis-driven proliferation of naive T cells [21, 27]. These immunological mechanisms could be ameliorated in CBT with low HLA disparity. However, associations between HLA disparity and the risk of immune reactions in CBT remain unclear [9, 11, 12, 16, 28].

Previous studies have reported a lower incidence of severe acute GVHD despite commonly used HLA 1 or 2 antigen-mismatched grafts in CBT compared with conventional allo-SCT [5, 13, 29]. However, the sample sizes in these studies were small (18 to 562 patients) and the incidences of grade II-IV acute GVHD varied widely from 26% to 51% [5, 6, 9–13, 15–17, 30]. Hence, further large-scale studies on acute GVHD after CBT are required. In addition, differences in the incidence of acute GVHD due to ethnicity, as in the case of chronic GVHD [19], also need to be investigated.

From Western countries, PIR after double-unit cord blood transplantation was reported [24, 25]. Recipients of double cord blood units experience grade II acute GVHD more frequently than single-unit recipients [24, 31], suggesting the difference of underlying cytokine profile. It is conceivable that the immune interaction between the two units may contribute to an enhanced immune reaction mediated by effector CD8^+^ T cells developing after CBT from naive precursors. Considering that cord blood grafts contain approximately 1-log fewer T cells, the vast majority of those cell are immunologically naive, there have been concerns regarding the ability of cord blood-derived lymphocytes to mediate graft-versus-malignancy effects. However, the incidence of leukemia recurrence following CBT is not different from that reported in recipients of bone marrow or peripheral blood SCT. Moreover, leukemia patients in remission who received myeloablative double unit CBT had less relapse rate [32]. Major or minor histocompatibility antigens expressed on hematopoietic stem cells of the losing unit might be shared by host leukemia cells, resulting in an enhanced graft-versus-malignancy (GVM) effects.

Brunstein et al. reported that KIR-ligand mismatch between the engrafted unit and the recipient had no favorable outcome in terms of acute GVHD, transplant-related mortality, and survival [33]. Even after reduced intensity conditioning, KIR-L mismatch resulted in significantly higher rates of grade III-IV acute GVHD and transplant-related mortality with inferior survival. Although ATG was incorporated only in one-third of patients, one could hypothesize that transplantation with KIR-L-mismatched CB units might result in better clinical outcomes, based on the premise that NK-cell alloreactivity dominates in the setting of low graft T-cell numbers. It is possible that the in vivo T-cell depletion secondary to ATG administration may have contributed to favor GVM effect in the presence of KIR-L mismatch. However, the impact of ATG on the outcomes of KIR-L-mismatched transplantations needs to be addressed in prospective studies.

### 2.2. Management of Early Immune Reactions

Optimal management of early immune reactions following CBT has not been established. In CBT incorporating ATG, in which immune reaction such as PIR or acute GVHD was reduced, infection is a primary cause of death with profound T-cell depletion in conjunction with delayed neutrophil recovery. CBT without ATG is associated with a significant risk of GVHD, and serious infections remain a challenge, especially in the setting of GVHD requiring systemic steroid. PIR itself will induce organ failure such as organ toxicity in the severe form [23], and the presence of PIR has been shown to cause more NRM, thus, preventive strategy or early intervention should be helpful [1]. 

It is to be noted that in several institute where more potent immunosuppressive regimen is routinely incorporated early posttransplant, PIR is not a major clinical issue after CBT [2, 13, 14, 34]. However, this does not warrant the intensification of immunosuppression for PIR like corticosteroids as preventative therapy after CBT, since there is concern that there may be increased risk of infection or relapse. Based on the experience of elderly patients who received *reduced*-intensity CBT, high early mortality related to PIR was substantially reduced by substituting Tacolimus for cyclosporine as GVHD prophylaxis [35]. Adding methotrexate [23, 36] or mycophenolate mofetile [37] may further improve the outcome. Recent study demonstrated that the strategic delivery of posttransplantation high-dose cyclophosphamide allows for the effective deletion of proliferating alloreactive cells in HLA-mismatched bone marrow transplantation. It may be postulated that cyclophosphamide selectively kills cells reactive to abundant alloantigens while minimally affecting cells reactive to tumor-specific antigens, and allowing the promotion of immunologic tolerance [38]. Although the type of graft is different, a strong but short-lived delivery of immune suppression may offer beneficial effect.

Once occurred, management of PIR in CBT is problematic. It is reasonable thought that the therapeutic goal will be immune suppression to an extent that PIR does not cause organ dysfunction. Early recognition of PIR and treatment with a short-course corticosteroid can help avoid unnecessarily long, empiric therapy that could promote opportunistic infections. Treatment of acute GVHD is similar to the treatment methods applied in the case of other grafts, such as SCT using bone marrow or peripheral blood stem cells. To the best of our knowledge, response to the treatment is relatively good. As previously described [14], some grade II-IV acute GVHD after CBT does not require systemic steroid administration. This is particularly true in Japanese adult CBT patients. In this regard, as well, it will be necessary to carry out large-scale validation studies in the future.

## 3. Chronic GVHD

### 3.1. Clinical Features

Comparative studies on unrelated bone marrow transplantation and CBT have shown that the incidence of chronic GVHD is lower in the *patients* who underwent CBT than those in bone marrow transplantation [5, 13, 39]. However, most of the studies related to chronic GVHD after CBT were small in scale and the reported incidences of chronic GVHD have shown great variation [5, 6, 9–18, 39–41]. The Japan Cord Blood Bank Network research on chronic GVHD in 2008, which is the largest study worldwide to date on this topic involved 1,072 patients [19]. In this large-scaled study, the incidence of post-CBT chronic GVHD was 28%, showing that, in Japan, the incidence of chronic GVHD *following CBT* is lower than the incidence of chronic GVHD following unrelated bone marrow transplantation [42, 43]. According to a recent report from Center for International Blood and Marrow Transplant (CIBMT), chronic GVHD *was* developed in 24% of patients, which was significantly lower compared with allele-matched *peripheral blood SCT*, and matched bone marrow transplantation [44]. In this retrospective study, 72% of CBT recipients received ATG. Regardless of graft source, the incidence of chronic GVHD was lower in those treated with ATG as part of the conditioning regimen. Thus, the result should be cautiously interpreted. ATG can provide potent immunomodulation and frequently incorporated towards improving transplant outcome. However, the role of ATG in CBT is still under debate.

Chronic GVHD following CBT is mild and responds well to treatment, as has been proved by earlier small-scale studies [10, 15–17, 40] and the large-scale study *from* our group in Japan [19]. The Japanese study found that the response rate for chronic GVHD was 68%, while the mortality rate was a mere 5% [19]. These rates are vastly superior to the comparative rates for bone marrow transplantation and peripheral blood SCT. These differences may contribute to favorable long-term immune recovery following CBT. Alternatively, mismatched unrelated cord blood may not have the potential to fully reconstitute immune function, especially in adult recipients. The impaired immune function may be attributed to decreased absolute immunocompetent cells in cord blood, disturbance of T-cell development in the host, reduced thymic output, and defects in B-cell antibody production [45, 46]. However, the differences inbasic mechanism *of* immune reconstitution between CBT and SCT using bone marrow or peripheral blood stem cells remain unclear and in need of further study.

In allogeneic SCT, the antitumor effect derives from both conditioning therapy prior to transplant and GVM effect arising from the attack of immunocompetent cells contained in the graft on the host's (patient's) malignant cells. As has been pointed out, in bone marrow transplantation, patients who develop mild chronic GVHD actually had a lower recurrence rate and improved outcomes [34, 47]. It was reported that chronic GVHD following CBT is also accompanied by a GVM effect [19]. In this study, multivariate analysis showed that the onset of chronic GVHD is a good prognostic factor not only for the disease-free survival rate but also the overall survival rate. At the same time, chronic GVHD was shown to be a significant factor in the prevention of recurrence and progression of the disease. The finding that chronic GVHD improves even the overall survival rate is noteworthy, since it contrasts with what occurs with bone marrow transplantation. In bone marrow transplantation, although chronic GVHD prevents recurrence and progression of the disease, some patients die due to the damages by chronic GVHD. Thus, the chronic GVHD itself can, in the end, offset the GVM effect, thus affecting the overall survival rate and the prognosis of the patient [34, 48, 49]. We surmise that these contrasting results are due to the fact that chronic GVHD following CBT is mild in severity compared to that occurring after bone marrow transplantation.

In reduced-intensity conditioning (RIC) CBT, mild severity of chronic GVHD observed in CBT may be translated into less GVM effect. In RIC-CBT, it was reported that leukemia-free survival was decreased, and relapse incidence was increased compared to those observed in myeloablative CBT. The patients who underwent RIC-CBT with acute myeloid leukemia in the second complete remission or the first complete remission duration <1 year had higher risk of relapse and poorer leukemia-free survival with similar incidence of chronic GVHD [50].

In Japan, our study group, the Nagoya Blood and Marrow Transplantation Group, carried out a small-scale study [20] on the clinical features of post-CBT chronic GVHD using an NIH criteria [51] *which is* originally developed for bone marrow transplantation and peripheral blood SCT. The severity of chronic GVHD is classified as mild, moderate, or severe on the basis of the target organ(s) and the clinical features. This study analyzed the data of 29 patients who survived for at least 100 days following CBT and found that chronic GVHD developed in 7 patients, with the limited type occurring in 3 patients who had lesions that were limited to part of the skin or only the liver, and the extensive type occurring in 4 patients who had lesions in multiple organs [20]. Using the NIH criteria, the chronic GVHD *following CBT* was classified as mild in 6 patients. The one remaining patient was classified as moderate GVHD. Thus, even by the NIH classification method, chronic *GVHD following *CBT was mild in severity, and the prognosis was indicated to be good.

### 3.2. Management of Chronic GVHD

Research [19, 20] conducted in Japan indicates that chronic GVHD following CBT has a lower incidence and is milder in severity compared with chronic GVHD following bone marrow transplantation and peripheral blood SCT, that it is also accompanied by a clinical GVM effect and that the demand for therapeutic intervention is lower than in the case of other grafts. Although, at present, chronic GVHD following CBT is generally treated by the same methods that were developed to manage chronic GVHD following bone marrow transplantation and peripheral blood SCT, it may be necessary to exercise caution with the initiation, intensity, and duration. The NIH evaluation method [51] may be a useful tool for making that judgment. Accordingly, in order to establish a recommended management system in the future, it will first be necessary to introduce the NIH's diagnostic and evaluation criteria, which are becoming ever more extensively used around the world for other transplantations, to the evaluation of chronic GVHD *following CBT*. Since it would be impractical to retrospectively rediagnose and reevaluate a large number of patients using those methods, it will be desirable to begin applying them to incoming patients. The data so generated would, then, probably enable effective investigation of the treatment methods.

## 4. Conclusion

Post-CBT immune reactions are unique. The fact that the clinical features of chronic GVHD, which is the major complication during long-term survival after allogeneic transplantation, are comparatively mild is very promising. If the short-term safety of CBT with regard to early immune reactions, and so forth, that are currently a special problem, could be improved, it would probably be possible to improve the overall results of CBT, making it a promising treatment for hematological diseases.
